# Ultrasonic-Cellulase Synergistic Extraction of Crude Polysaccharides from *Moringa oleifera* Leaves and Alleviation of Insulin Resistance in HepG2 Cells

**DOI:** 10.3390/ijms232012405

**Published:** 2022-10-17

**Authors:** Fan Gu, Liang Tao, Runling Chen, Jiao Zhang, Xingzhong Wu, Min Yang, Jun Sheng, Yang Tian

**Affiliations:** 1 College of Food Science and Technology, Yunnan Agricultural University, Kunming 650201, China; 2 National Research and Development Professional Center for Moringa Processing Technology, Yunnan Agricultural University, Kunming 650201, China; 3 Engineering Research Center of Development and Utilization of Food and Drug Homologous Resources, Ministry of Education, Yunnan Agricultural University, Kunming 650201, China; 4 Yunnan Provincial Engineering Research Center for Edible and Medicinal Homologous Functional Food, Yunnan Agricultural University, Kunming 650201, China

**Keywords:** *Moringa oleifera* leaves crude polysaccharides, ultrasonic-cellulase extraction, response surface methodology, antioxidant, insulin resistance

## Abstract

*Moringa oleifera* leaves (MOL) are a new food resource, rich in functional factors. MOL polysaccharides are important active macromolecules within MOL. However, there are problems, such as low extraction rates and lack of evidence for functional activity. Therefore, in this experiment, single-factor experiments were carried out using MOL powder as the raw material, and the Plackett–Burman test was used to screen the significantly influential test factors. The extraction process of MOL polysaccharide was optimized by response surface methodology. The insulin resistance alleviating activity of MOLP polysaccharides was initially explored. The results showed that the extraction of *Moringa oleifera* leaves crude polysaccharides (MOLP) by ultrasonic assisted cellulase enzymatic digestion was (17.03 ± 1.03)%, and the obtained MOLP was a crude polysaccharide with an average molecular weight (Mw) of 279.48 kDa, consisting of fucose, rhamnose, arabinose, galactose, glucose, xylose, mannose, galacturonic acid, and glucuronic acid. MOLP had an IC50 value of 8.02 mg/mL for α-glucosidase and scavenging activity against free radicals such as ABTS, DPPH, hydroxyl radicals, and superoxide anion with an IC50 value of 0.21 mg/mL 0.31 mg/mL 0.97 mg/mL 0.49 mg/mL. At the same time, MOLP significantly enhanced the glucose consumption, glycogen synthesis, CAT, SOD, GSH-Px activity, and reduced the MDA and ROS content in high glucose-induced insulin-resistant HepG2 (IR-HepG2) cells. This experiment improved the extraction rate of MOLP and demonstrated that MOLP has antioxidant activity and α-glucosidase inhibitory activity, which can alleviate the insulin resistance of high glucose-induced HepG2 cells. It provides partial data support for the possible hypoglycemic effect of MOLP by alleviating oxidative stress, and also provides new ideas for the in-depth study of basic research and industrial application of MOLP.

## 1. Introduction

Diabetes mellitus (DM) is the third most common chronic non-communicable disease after cancer and cardiovascular disease, posing a serious threat to human health, especially type 2 diabetes mellitus (T2 DM), which accounts for 90–95% of all diabetics and has developed into a global public health problem [1]. DM is a complex metabolic disorder characterised by hyperglycaemia and impaired pancreatic beta-cell function caused by insulin resistance (IR). IR is a condition of impaired glucose utilization and decreased insulin sensitivity, manifested by the inability of normal circulating concentrations of insulin to effectively stimulate glucose uptake and utilization in target tissues including skeletal muscle, liver, and adipose tissue [2].

Oxidative stress is defined as an imbalance between the production of reactive oxygen species (ROS) and antioxidant defense mechanisms. There is also growing evidence that oxidative stress may be an important factor in hyperglycaemia-induced insulin resistance in T2 DM. Mitochondrial dysfunction, overproduction of reactive oxygen species (ROS) and lipid peroxidation were found in the livers of Zucker rats with T2 DM [3]. Increased oxidative stress may be a harmful factor contributing to insulin resistance and impaired glucose tolerance in patients with type 2 diabetes. Chen et al. [4] showed that apple pomace polysaccharides protect cells from palmitate-induced insulin resistance and loss of viability by inhibiting mitochondrial ROS and rescuing mitochondrial respiratory function. In an in vitro antioxidant and antidiabetic study of Schisandra sphenanthera polysaccharide in type 2 diabetic rats, it was shown that Schisandra sphenanthera polysaccharide had a hypoglycaemic effect on diabetic rats, the mechanism of which may be related to its antioxidant effect [5]. Tartary buckwheat flavonoids protect hepatocytes against high glucose-induced oxidative stress and insulin resistance through MAPKs signalling pathway [6]. In summary, the alleviation of oxidative stress can be a potential therapeutic strategy to prevent or delay insulin resistance diseases such as diabetes. The liver is one of the important target organs for insulin action and plays a crucial role in controlling glucose homeostasis [7]. Improving hepatic IR is considered an important strategy for the treatment of T2 DM and is of great importance.

*Moringa oleifera* Lam is a perennial tropical deciduous tree in the genus *Moringa*, a medicinal and food plant [8], which was approved as a new resource food by the Chinese Ministry of Health in 2012. It is also known as the “drumstick tree” because of the pungent smell of its roots, and the trunk resembles a drumstick [9]. However, the main medicinal and edible part of *Moringa oleifera* is the *Moringa oleifera* leaves (MOL), which are rich in polysaccharides, proteins, polyphenols, and other nutrients [10]. MOL aqueous extracts have biological activities such as antioxidant [11], hypoglycaemic, antibacterial, and anti-inflammatory [12]. Polysaccharides are another class of biomolecules other than proteins and nucleic acids that can maintain the normal functioning of the body. They are gradually becoming a research hotspot in the field of biochemistry and molecular biology because of their efficacy in antioxidant [13], antitumour [14], hypoglycaemic [15], and hypolipidemic [16]. MOLP is one of the plant-derived polysaccharides with good water solubility and is an important active ingredient in the aqueous extract of MOL, with the content ranging from about 8.61% to 33.61% [17]. It has been shown that *Moringa oleifera* leaves polysaccharides have immune-enhancing activity [18] and prevent obesity by affecting the gut microbes [19]. The flavonoid complex of *Moringa oleifera* leaves polysaccharide has hypoglycaemic effects [20]. However, few studies have shown that MOLP reduces insulin resistance by alleviating oxidative stress.

The objectives of this study were three-fold: (1) to improve the extraction rate of MOLP; (2) to discover an alpha-glucosidase inhibitor, and (3) to clarify that MOLP alleviates insulin resistance by reducing oxidative stress. This study may provide a theoretical basis for the reduction of insulin resistance by polysaccharides through the alleviation of oxidative stress, and provide data to support further in vivo demonstration of the potential hypoglycaemic activity of MOLP.

## 2. Results

### 2.1. Ultrasound-Assisted Cellulase Extraction of Polysaccharides from MOL Single-Factor Experiment

**Figure 1:**
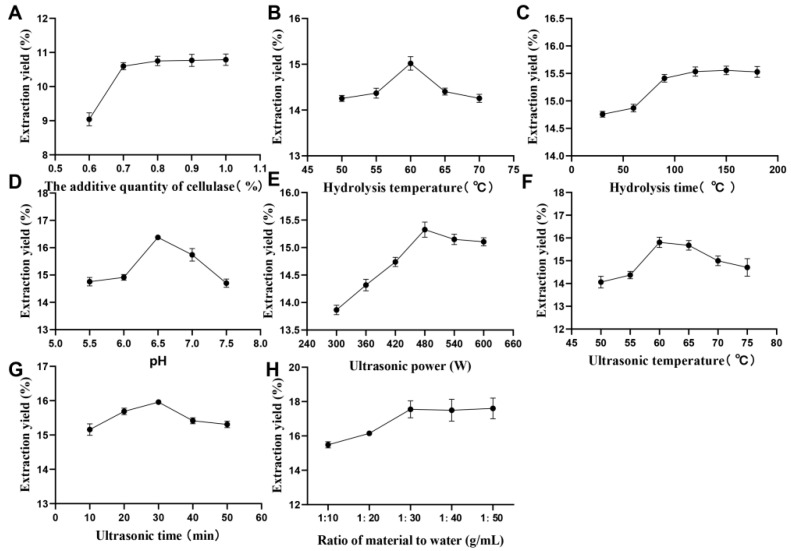
Ultrasonic-assisted cellulase extraction of MOLP in a single-factor experiment. (**A**) Effect of cellulase addition on the extraction rate of MOPL; (**B**) Effect of hydrolysis temperature on the extraction rate of MOLP; (**C**) Effect of hydrolysis time on the extraction rate of MOLP; (**D**) Effect of pH on the extraction rate of MOLP; (**E**) Effect of ultrasonic power on the extraction rate of MOLP; (**F**) Effect of ultrasonic temperature on the extraction rate of MOLP; (**G**) Effect of ultrasonic time on the extraction rate of MOLP; (**H**) Effect of the material-to-water ratio on the extraction rate of MOLP.

The impact of cellulase addition on the rate of polysaccharide extraction is depicted in Figure 1A. The results show that the extraction rate of polysaccharides showed a trend of first increasing and then levelling off with the increase of cellulase addition, and the extraction rate of polysaccharides from MOL reached a maximum of (10.75 ± 0.14)% with an addition amount of 0.8%. The highest extraction rate was obtained at a hydrolysis temperature of 60 °C, as shown in Figure 1B. According to Figure 1C, the optimal hydrolysis time is 120 min. Figure 1D shows that the extraction rate of MOL polysaccharides shows a trend of increasing and then decreasing with an increasing pH value. The highest extraction rate, 16.38 ± 0.34% of MOLP, was obtained when the pH was 6.5. 

Figure 1E shows that a 480 W ultrasonic power setting resulted in the maximum polysaccharide extraction rate. As observed in Figure 1F, the ultrasonic temperature of 60 °C produced the maximum extraction rate of polysaccharide. From Figure 1G, it can be seen that the highest extraction rate of polysaccharide was achieved when the ultrasonic time was 30 min. As can be seen from Figure 1H, the highest polysaccharide extraction rate of 17.55 ± 0.49% was achieved when the material-to-water ratio was 1:30.

### 2.2. Plackett–Burman Experiment

The factors that have a greater effect on the extraction rate of MOLP were screened from eight factors using PB tests. The results of the PB experiment are shown in Table 1 and the Plackett–Burman test ANOVA is shown in Table 2.

Table 2 shows that the following four single factors had a significant effect on the extraction rate of MOLP: The additive quantity of cellulase, hydrolysis temperature, ultrasonic power, and ultrasonic time.

### 2.3. Response Surface Optimisation of the Extraction Process

**Figure 2:**
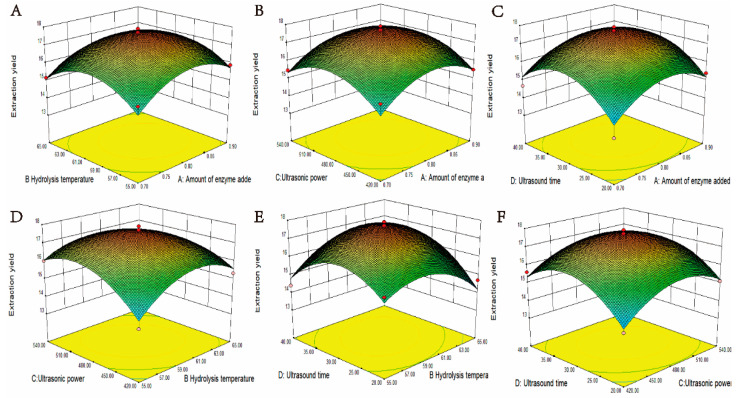
Response surface diagram of the interaction between the factors. (**A**) Effect of hydrolysis temperature and enzyme addition on the extraction rate of the polysaccharides. (**B**) Effect of ultrasonic power and enzyme addition on the extraction rate of the polysaccharides. (**C**) Effect of ultrasonic time and amount of enzyme addition on the extraction rate of the polysaccharides. (**D**) Effect of ultrasonic power and hydrolysis temperature on the extraction rate of the polysaccharides. (**E**) Effect of ultrasonic time and extraction rate of hydrolysis temperature on the extraction rate of the polysaccharides. (**F**) Effect of ultrasonic time and ultrasonic power on the extraction rate of the polysaccharides.

The BoxBehnken test was performed to obtain the optimal combination of parameters for the extraction of MOLP. Based on the results of the above single-factor experiment and PB test, 29 sets of experiments with 4 factors and 3 levels were designed, and the results of the response surface are shown in Table 3 and the ANOVA table is shown in Table 4. A 3D plot of the effect between the two factors on the extraction rate of MOLP is shown in Figure 2. The logical relationship between the response values and the tested variables is analysed using the Design-Expert software. The multivariate quadratic equation for the extraction rate of MOLP was obtained after analysis.

*Z* = 17.57 + 0.32*A* + 9.783*E* − 003*B* + 0.24*C* + 0.31*D* − 0.092*AB* − 0.028*AC* − 0.056*AD* − 0.50*BC* + 0.56*BD* − 0.17*CD* − 1.07*A*^2^ − 1.15*B*^2^ − 1.10*C*^2^ − 1.36*D*^2^, (1)

where *Z* represents the extraction rate of MOLP, *A* represents the amount of enzyme added, *B* represents the hydrolysis temperature, *C* represents the ultrasonic power, and *D* represents the ultrasonic time.

As can be seen from Table 4, the regression model is *p* < 0.0001 (highly significant) and the misfit term *p* = 0.1796 > 0.05 (not significant), indicating that the regression model is reliable and can be used for the optimization of the polysaccharide extraction rate of MOL. In addition, the coefficient of determination of the model, *R*^2^ = 0.897, indicates a good fit. Therefore, the regression model can be used to analyse and predict the extraction rate of MOLP. As can be seen from the F-values, the effect of each factor in the range on the yield of MOLP is in the following order: amount of enzyme added > hydrolysis time > hydrolysis temperature > ultrasonic power.

As can be seen from Figure 2E, the response surface slopes steeply and the interaction between ultrasonic time and hydrolysis temperature is significant. Among the four factors, the amount of enzyme added and the time of ultrasonic had the greatest effect on the extraction rate of MOLP. In addition, the extraction rate of polysaccharides increased rapidly with the increase of enzyme addition and sonication time.

The extraction rate of MOLP was predicted by Design-Expert software at 0.81% enzyme addition, 60.01 °C hydrolysis temperature, 486.04 W ultrasonic power, and 31.06 min ultrasonic time to be 17.623%. For practical applications, the parameters were adjusted to 0.80% enzyme addition, 60.00 °C hydrolysis temperature, 490 W ultrasonic power, and 30 min ultrasonic time for validation experiments to obtain a polysaccharide extraction rate of (17.03 ± 1.03)%. The difference between the predicted extraction rate and the actual extraction rate was small. Therefore, response surface analysis was used to optimize the extraction process of MOLP, and the results were accurate and reliable. Therefore, the best extraction process for MOLP was obtained with an enzyme addition of 0.80%, a hydrolysis temperature of 60 °C, a hydrolysis time of 120 min, a pH of 6.0, an ultrasonic power of 490 W, an ultrasonic temperature of 60 °C, an ultrasonic time of 30 min, and a material-to-water ratio of 1:30.

### 2.4. Inhibition of α-Glucosidase and Antioxidant Activity of MOLP

**Figure 3:**
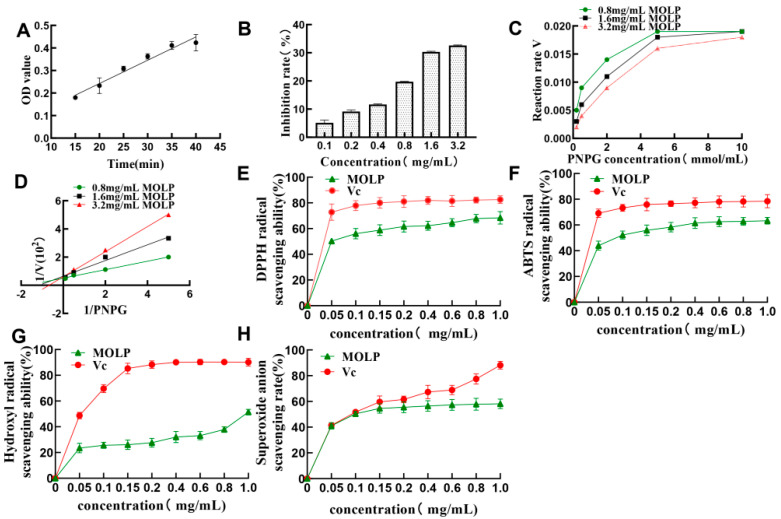
MOLP effects on α-glucosidase and free radical scavenging activity. (**A**) α-glucosidase and PNPG reaction times; (**B**) Effect of MOLP on α-glucosidase. (**C**,**D**) Kinetic analysis of the inhibitory effect of MOPL on α-glucosidase; (**E**) Effect of MOPL on DPPH radical scavenging activity. (**F**) Effect of MOPL on the scavenging activity of ABTS radicals; (**G**) Effect of MOPL on the scavenging activity of hydroxyl radicals; (**H**) Effect of MOPL on the scavenging activity of superoxide anion radicals. Data are expressed as the mean ± SD of three independent experiments (n = 3).

As can be seen from Figure 3A, the absorbance value of the reaction system (at 405 nm) increased with time. When the reaction time was between 15 and 40 min, the reaction between enzyme and substrate showed a relatively good linear relationship with time. At 40 min, the growth trend becomes slower. Therefore, the reaction between α-glucosidase and PNPG was more complete at 35 min and the optimum reaction time between enzyme and substrate was chosen to be 35 min. As shown in Figure 3B, the MOLP showed good inhibitory activity against α-glucosidase, where the inhibition rate increased with the increasing polysaccharide concentration, with an IC50 value of 8.02 mg/mL. This indicates that MOLP has an inhibitory effect on α-glucosidase.

To determine the type of inhibition of α-glucosidase by MOLP, the relationship between MOLP concentration, substrate concentration, and enzymatic digestion rate was investigated according to the Lineweaver–Burk double inverse curve plotting method to determine the type of inhibition. As can be seen from Figure 3C, the rate of the enzymatic reaction increases with the increasing substrate concentration and gradually approaches the maximum reaction rate of Vmax. This result suggests that MOLP are able to compete for substrates and preferentially bind to enzymes to form complexes, thereby inactivating them [21]. Figure 3D shows the Lineweaver–Burk plot, from which it can be seen that the slope of the straight line increases with the increasing concentration of MOLP, and the intersection of the straight lines of different concentrations of polysaccharide is almost close to the y-axis. This characteristic is consistent with competitive inhibition, and therefore MOLP are competitive inhibitors.

The scavenging rates of ABTS, DPPH, hydroxyl radicals, and superoxide anions by MOLP are shown in Figure 3. The scavenging activity of MOLP showed an IC50 value of 0.21 mg/mL for ABTS, 0.31 mg/mL for DPPH, 0.97 mg/mL for superoxide anion, and 0.49 mg/mL for hydroxyl radicals, showing an increasing trend with increasing concentrations.

### 2.5. MOLP Attenuates High Glucose-Induced Oxidative Stress in IR-HepG2 Cells

**Figure 4:**
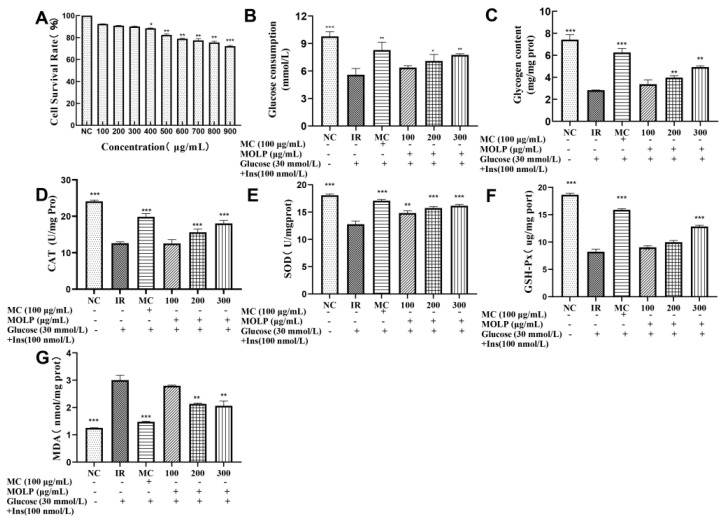
Effect of MOLP on high glucose-induced IR-HepG2 cells. (**A**) Effect of MOLP on the viability of HepG2 cells. (**B**) Effect of MOLP on glucose consumption in IR-HepG2 cells. (**C**) Effect of MOLP on glycogen synthesis in IR-HepG2 cells. (**D**) Effect of MOLP on CAT activity of IR-HepG2 cells. (**E**) Effect of MOLP on SOD activity of IR-HepG2 cells. (**F**) Effect of MOLP on GSH-Px activity of IR-HepG2 cells. (**G**) Effect of MOLP on MDA content of IR-HepG2 cells. Data are expressed as mean ± SD of three independent experiments (n = 3), compared to the IR group * *p* < 0.05, ** *p* < 0.01, *** *p* < 0.001. (NC indicates normal control group, IR indicates insulin resistant group, MC indicates positive control group (metformin control), 100 indicates 100 μg/mL MOLP treatment group, 200 indicates 200 μg/mL MOLP treatment group, 300 indicates 300 μg/mL MOLP treatment group).

The effect of MOLP on high glucose-induced IR-HepG2 cells is shown in Figure 4. First, the effect of MOLP on the survival rate of HepG2 cells was investigated (Figure 4A). The results showed that MOLP had no growth-promoting effect on HepG2 cells and showed a decreasing trend in the survival rate of HepG2 cells as the concentration of MOLP increased. The cell survival rate was (92.40 ± 0.15% − 90.83 ± 0.10)% in the range of 100–300 μg/mL compared to the control group, with no toxic effect on the cells (>90%). When the MOLP concentration reached 400 μg/mL, it was significantly reduced compared to the control group and had a toxic effect on the cells. On balance, 100, 200, and 300 μg/mL were chosen for the experiment. 

Next, the alleviating effect of MOPL on insulin resistance was studied (Figure 4B,C). The results showed a significant increase in glucose consumption after MOLP treatment at polysaccharide concentrations of 200 μg/mL (7.10 ± 0.82 mmol/L) and 300 μg/mL (7.75 ± 0.14 mmol/L) under treatment in the dose group. The glycogen content of IR-HepG2 cells was 3.97 ± 0.19 mg/mg prot at 200 μg/mL MOLP treatment and 4.92 ± 0.12 mg/mg prot at 300 μg/mL MOLP treatment, with increased glycogen content compared to the insulin-resistant group. 

The final study of MOLP alleviated oxidative stress in IR-HepG2 cells (Figure 4D–G). The results showed that high concentrations of glucose caused significantly lower GSH-Px, CAT, and SOD activities than the control group (Figure 4D–F). MOLP treatment of IR-HepG2 cells increased GSH-Px, CAT, and SOD activities to different degrees and reduced the MDA content.

### 2.6. Effect of MOLP on the ROS Content of IR-HepG2 Cells

**Figure 5:**
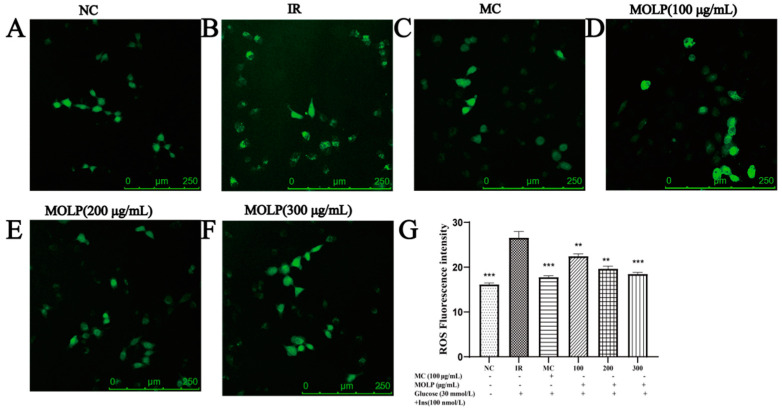
Effect of MOLP on ROS in IR-HepG2 cells. (**A**–**F**) Pictures were taken by laser scanning confocal microscopy; (**G**) The average fluorescence intensity was analysed by Image J. Data are expressed as mean ± SD of three independent experiments (n = 3), compared to the IR group ** *p* < 0.01, *** *p* < 0.001. (NC indicates normal control group, IR indicates insulin resistant group, MC indicates positive control group (metformin control), 100 indicates 100 μg/mL MOLP treatment group, 200 indicates 200 μg/mL MOLP treatment group, 300 indicates 300 μg/mL MOLP treatment group).

The effect of MOLP on the fluorescence intensity of ROS in IR-HepG2 cells is shown in Figure 5. Compared with the control group, the fluorescence intensity was relatively high in the model group (26.59 ± 0.67) and significantly lower in intracellular ROS fluorescence intensity after MOLP treatment (22.47 ± 0.55, 19.67 ± 0.37, 18.47 ± 0.26), where the fluorescence intensity at a polysaccharide concentration of 300 μg/mL was close to that of the positive control metformin group (17.79 ± 0.28). This suggests that the MOLP can effectively reduce the fluorescence intensity of ROS in IR-HepG2 cells and may protect IR-HepG2 cells from free radical damage by reducing the amount of ROS.

## 3. Discussion

With rising living standards and the increased intake of high-calorie, high-sugar foods, the number of people with diabetes is increasing worldwide every year. Preventing diabetes and treating people with pre-diabetes is an important way to reduce the number of people with diabetes. Medication to control the progression of the disease is not without side effects, and natural plants and herbs with hypoglycaemic activity are popular with patients. MOL are the main active part of *Moringa oleifera* Lam, available as food and medicine. Not only does it have biological activities such as antioxidant and hypoglycaemic, but it has also shown outstanding results in alleviating the complications of diabetes. Pre-diabetic patients who consumed 6 × 400 mg of dried *Moringa oleifera* leaves powder capsules per day for 12 weeks showed a decrease in TNF-α in their bodies [22]. Methanolic extract from *Moringa oleifera* leaves promotes wound healing in diabetic rats [23]. *Moringa oleifera* leaf aqueous extract prevents other metabolic changes and complications associated with the hyperglycaemic state when administered to diabetic animals under acute conditions for prolonged periods of time [24]. MOL have shown interesting results both in terms of lowering blood sugar and alleviating diabetic complications. MOLP is an important active molecule in the aqueous extract of MOL. This has made it possible to study the hypoglycaemic activity of Morinda citrifolia polysaccharides.

In this study, ultrasonic-assisted cellulase was used to extract MOLP, and the extraction rate of MOLP was successfully improved. The aim was to investigate the antioxidant capacity, α-glucosidase inhibitory activity, and the ability to alleviate insulin resistance in HepG2 cells. The results showed that MOLP had inhibitory activity against α-glucosidase, alleviated oxidative stress in high glucose-induced insulin-resistant HepG2 cells, and attenuated the continued development of insulin resistance.

Extraction methods are an important factor in the use of active plant components [25]. In recent years, hot water extraction (HWE), ultrasonic assisted extraction (UAE), and cellulase extraction (CE) have been commonly used to extract plant polysaccharides. HWE is a traditional extraction method that usually requires higher extraction temperatures and longer extraction times, but has low extraction yields [26]. UAE had been widely used to obtain active components from different plant materials with a minimal impact on biological activity owing to its cell disruption, improved penetration, and capillary effects [27,28]. In addition, the high-frequency oscillation, cavitation, and mechanical shear of the ultrasonic treatment enhance the mass transfer between the solid particles and the liquid, thus promoting the diffusion of the extracted liquid in the direction of the liquid phase [27,29]. However, the higher power of ultrasonic and the longer extraction time tend to destroy the active ingredients. CE can disrupt the dense structure of the cell wall and accelerate the dissolution of polysaccharides, thus increasing the extraction rate, but at a higher cost. Ultrasound-Cellulase Synergistic Extraction (UCSE) is a new combined technology of UAE and CE that can make full use of each other’s advantages. It overcomes the disadvantages of traditional extraction methods and effectively increases the extraction rate of polysaccharides. This study showed that ultrasonic-assisted cellulase extraction of MOLP resulted in a polysaccharide extraction rate of 17.03 ± 1.03%. This was higher than the extraction rate of *Moringa oleifera* leaves polysaccharide (16.24%) by Sui et al. [30], who used ultrasonic assisted ultra-high pressure extraction, which was higher than the extraction rate of *Moringa oleifera* leaves polysaccharide (7.39%) by Zhang et al. [17], who only used ultrasonic assisted extraction. In summary, cellulase may play an important role in the extraction of MOLP, and there is a synergistic effect between cellulase and ultrasound, which effectively increases the extraction rate of MOLP. From Figure 1A–D, it can be seen that the extraction rate of MOLP is mainly caused by the influence of cellulase properties. In Figure 1E,G, the extraction rate of MOLP slightly decreased after reaching the maximum, which may be due to the multiple effects caused by excessive ultrasonic extraction, which may disrupt the chain structure of polysaccharides, leading to the breakdown of the polysaccharide spatial structure and the formation of small molecular fragments [31]. At the same time, the extraction rate of MOLP decreased as the ultrasonic temperature increased (Figure 1F), which may be due to the effect of extraction temperature on the effect of ultrasonic cavitation. As the system temperature increases, the maximum temperature and maximum pressure of the cavitation bubbles produced by ultrasonic disintegration also decreases. Excessively high system temperatures reduce the energy generated during cavitation bubble rupture, reducing the cavitation effect and thus the extraction capacity [32]. 

Improving the extraction rate of MOLP is the basis for studying the biological activity of MOLP. Studying the potential hypoglycaemic effect of MOLP is an important way to increase its application value. α-glucosidase is a digestive enzyme that plays a very key role in the metabolism of sugars, mainly by hydrolysing the non-reducing end of the polysaccharide’s α-1,4 glycosidic bond or oligosaccharide, releasing free glucose. Normally, starchy foods are hydrolysed to oligosaccharides by saliva and pancreatic α-amylase. It is further hydrolysed to glucose by α-glucosidase in the small intestine [33]. Therefore, the search for drugs that effectively inhibit α-glucosidase activity is of great importance in regulating blood glucose concentration and controlling postprandial hyperglycaemia. Therefore, the search for drugs that effectively inhibit α-glucosidase activity is important for regulating blood glucose concentration and controlling postprandial hyperglycaemia. The type of inhibition of α-glucosidase by MOLP was competitive and it was consistent with the type of inhibition by maize silk polysaccharide [34]. The IC50 value of MOLP for α-glucosidase inhibition was 8.02 mg/mL, which was higher than that of the WXA-1 fraction of wheat bran polysaccharide studied by Lv et al. [35] for α-glucosidase inhibition (IC50 = 1.17 mg/mL) and higher than that of papaya seed polysaccharide studied by Deng et al. [36], whose IC50 value was 6.24 mg/mL. This may be due to the fact that MOLP is a crude polysaccharide, and further purification is needed to improve the inhibitory effect of MOLP on α-glucosidase and to clarify the inhibitory component of MOLP on α-glucosidase. In addition, MOLP do not inhibit α-glucosidase at high rates and other regulatory mechanisms may be present.

Among the various ROS, the most chemically active hydroxyl radicals can cause damage to biomolecules such as proteins and nucleic acids [37]. Superoxide anion radicals are relatively weak oxidants and the most common free radicals produced in the body, and they are one of the precursors of superoxide anion radicals and hydroxyl radicals that can cause tissue damage [38]. Li et al. [39] have reported that excess superoxide anion radicals are thought to be the beginning of ROS accumulation in cells, leading to redox imbalance and associated harmful physiological consequences. DPPH radicals are stable free radicals and the assessment of the scavenging activity of DPPH is a rapid and effective way to detect antioxidant activity [40]. Therefore, the search for a natural compound with good scavenging ability against hydroxyl radicals, DPPH radicals, and superoxide anion radicals is important for the treatment and prevention of diseases caused by ROS. Studies have shown that pumpkin polysaccharides can inhibit carbohydrate digestive enzymes, DPPH, and ABTS radicals. It also protects pancreatic β-cells from oxidative damage by reducing MDA levels and increasing SOD activity. Thus, its hypoglycaemic potential has been revealed [41]. In the evaluation of the antioxidant capacity of MOLP, it was found that it had a stronger scavenging effect on ABTS, hydroxyl radicals, DPPH, and superoxide anion radicals, which was higher than that of isolated and purified red pine cone polysaccharide [42] against hydroxyl radicals (IC50 = 3.0 mg/mL), ABTS radicals (IC50 = 23.6 mg/mL). It also has higher scavenging activity than *Dryopteris crassirhizoma* Nakai polysaccharides [43] against DPPH radicals (IC50: 2.04 mg/mL), hydroxyl radicals (IC: 1.70 mg/mL), and superoxide anions (IC50: 4.20 mg/mL). A large number of studies have shown a correlation between oxidative stress and hypoglycaemia. The MOLP in this study had strong free radical scavenging activity, so the next step was to establish an insulin resistance model using high sugar medium, aiming to reveal the potential hypoglycaemic effect of MOLP from the perspective of reducing oxidative stress.

The liver plays an important regulatory role in maintaining dynamic glucose homeostasis. Following an oral glucose load, one-third of the glucose is absorbed by the liver [44]. HepG2 is an immortalised cell line that is widely used in biochemical and nutritional studies because it maintains a high level of morphological differentiation and function in vitro [45]. Therefore, HepG2 cells are suitable for in vitro studies of glucose production and insulin pathway regulation [46]. Oxidative stress is thought to be a major cause of IR development, with high concentrations of glucose in cells leading to excessive accumulation of ROS and ultimately to oxidative stress [47]. Changes in antioxidant enzyme activity are biomarkers of the antioxidant response. Superoxide dismutase (SOD) and catalase (CAT) are the main antioxidant enzymes that protect cells from harmful effects mediated by ROS. GSH-Px plays an important role in protecting cell membranes from lipid peroxidation damage [48]. Therefore, the activity of these antioxidant enzymes is essential for the quenching of intracellular peroxides in cell destruction and for inhibiting the cytotoxicity of ROS overproduction. On this basis, we measured the activity of GSH-Px, CAT, SOD, and the content of MDA and ROS. The results showed that the MOLP group could effectively enhance GSH-Px, CAT, and SOD activities (*p* < 0.05) and reduce MDA (Figure 4) and ROS content (Figure 5), compared to the model group. Similar results have been reported by Chai et al. [49] who found that CAP2-1 polysaccharide could directly scavenge ROS by significantly enhancing the content of the antioxidant enzyme SOD and the non-enzymatic antioxidant GSH in oxidatively damaged cells. It improved cell viability and membrane integrity, enhanced intracellular antioxidant capacity, and reduced oxidative stress. These results suggest that high doses of glucose can induce insulin resistance in HepG2 cells and that MOLP may protect IR-HepG2 cells from oxidative stress by activating antioxidant enzymes to inhibit intracellular ROS, thus achieving relief of insulin resistance. However, the present study is only a preliminary indication that MOLP can enhance glucose consumption, glycogen synthesis, and antioxidant enzyme activity in IR-HepG2 cells, with potential hypoglycaemic effects. MOLP is a crude polysaccharide, and it still needs to be further isolated and purified to produce an active ingredient with a high level of insulin resistance relief. Transcriptomics techniques are being used to continue to investigate the mechanisms by which MOLP are regulated at the cellular level and to clarify whether they alleviate oxidative stress by activating antioxidant signalling pathways, thereby reducing insulin resistance. After demonstrating the role of MOLP in alleviating insulin resistance at the cellular level, animal experiments will continue to be used to verify whether the signalling pathways regulated by MOPL are consistent with the cellular level, and to clarify whether the mechanism of MOPL regulation in vivo is the same as at the cellular level.

## 4. Materials and Methods

### 4.1. Materials

*Moringa oleifera* leaves powder was provided by Yunnan Dehong Tianyou Biotechnology Co., Ltd. (Dehong, China). Cellulase (C8270), D-glucose anhydrous standard (G8150), disodium hydrogen phosphate (D7292-50g), sodium dihydrogen phosphate (S5830), bovine serum albumin (A8010), p-nitrophenyl-a-D-glucopyranoside (PNPG, N8700), α-glucosidase (G8823), salicylic acid (S7080), 1,1-Dimethylbiguanide hydrochloride (Metformin, D9351), CAT (BC0200), GSH-Px (BC1190), and MDA (BC0025) kits were purchased from Solarbio Science & Technology Co., Ltd. (Beijing, China). Concentrated sulfuric acid was purchased from Chongqing East Chemical Co., Ltd. (Chongqing, China). Chloroform was purchased from Chengdu Kelong Chemical Co., Ltd. (Chengdu, China); HepG2 hepatocellular carcinoma cells were obtained from Kunming Cell Bank of the Chinese Academy of Sciences. (Kunming, China). 1,1-diphenyl-2-picrylhydrazyl (DPPH, 257621), Dulbecco’s Modified Eagle’s Medium (DMEM, D0819) were purchased from Sigma (Sigma–Aldrich Chemical Co., St. Louis, MO, USA). The SOD (A001-3-2) test kit, Glucose Assay Kit (F006-1-1) and glycogen assay kit (A043-1-1) were obtained from Nanjing Jiancheng Biological Engineering Institute. (Nanjing, China). ABTS (A276045), phenol (P128316-500g), anhydrous ethanol (E118433-5L), n-butanol (B119677), and anhydrous sodium carbonate (S111733-100g) were purchased from Aladdin Trading Co., Ltd. (Shanghai, China). The Reactive Oxygen Assay Kit (BL714A) was purchased from biosharp Biotechnology Ltd. (Beijing, China)

### 4.2. Optimization of the Extraction Process of MOLP

We improved the method of polysaccharide extraction by Chen et al. [50]. First, we weigh 5 g of *Moringa oleifera* leaves powder, add the corresponding volume of water according to the ratio of material to liquid, stir well, adjust the pH of the solution, and add cellulase. After soaking in a set constant temperature water bath, the enzyme was inactivated at 88 °C for 10 min and then sonicated in an ultrasonic apparatus at the set sonication power and temperature. The single-factor experimental design and the single-factor order in which the experiments were conducted are shown in Table 5. The experiments were conducted sequentially, changing one factor at a time.

The aqueous extract of *Moringa oleifera* leaves was removed from the precipitate by filtration and the polysaccharide content was determined by the phenol-sulphuric acid method. The glucose standard curve is shown in the attached Figure S1. Plackett–Burman (PB) experiments were performed using Design-Expert.V8.0.6.1 software based on the results of the single factors, with the aim of screening out the factors with significant effects from the eight factors, followed by Box–Behnken response surface optimisation tests. The PB experimental design factor level table is shown in Table S1 and the response surface experimental design factor level table is shown in Table S2. MOLP extraction % = (*Moringa oleifera* leaves aqueous extract polysaccharide content/*Moringa oleifera* leaves powder weight) × 100%.

### 4.3. Preparation of Crude Polysaccharides from Moringa oleifera Leaves (MOLP)

*Moringa oleifera* leaves powder was extracted using the optimal extraction process to obtain *Moringa oleifera* leaves crude polysaccharide extract. The extract was concentrated to one-quarter as in the study by Dong et al. [51] and defatted using petroleum ether. AB-8 macroporous sorbent resin was added to the extract and shaken to remove the pigments, followed by 12 times deproteinization of the extract using the Sevage method. Finally, the extract was dialyzed for 48 h using a 3000 Da dialysis bag. Four times the volume of anhydrous ethanol was added to the extract and left to stand overnight at 4 °C. The sample was centrifuged (4000 r/min) for 15 min and freeze-dried under a vacuum to obtain MOLP. The monosaccharide composition of the MOLP is shown in Table S3 and the molecular weights are shown in Figure S2.

### 4.4. Determination of Antioxidant Activity

#### 4.4.1. Determination of DPPH Radical Scavenging Capacity

The ability of MLPH to scavenge DPPH radicals was investigated using the method of Wang et al. [52] with slight modifications. An ethanol solution of 0.1 mmol/L DPPH was prepared by weighing 4 mg of DPPH dissolved in 100 mL of anhydrous ethanol, placed in a refrigerator at 4 °C, and stored in a brown bottle sealed from light. The absorbance values of 1 kDa, 3 kDa, 5 kDa, and 10 kDa samples were measured by taking 0.5 mL of the solution to be measured and 0.5 mL of 0.1 mmol/L ethanol solution in a 2 mL centrifuge tube, protected from light for 50 min, and centrifuged (10 min 4000 r/min) at a wavelength of 517 nm.

(2) $$\mathrm{DPPH}\;\mathrm{radical}\;\mathrm{scavenging}\;\mathrm{activity}\;(\%)=\frac{A_{1}-(A_{2}-A_{3})}{A_{1}}\times 100,$$

where *A*_1_ is anhydrous ethanol instead of 0.1 mmol/L DPPH; *A*_2_ is 0.5 mL of anhydrous ethanol and 0.5 mL of 0.1 mmol/L DPPH in ethanol solution; and *A*_3_ is 0.5 mL of the sample and 0.5 mL of 0.1 mmol/L DPPH in ethanol solution.

#### 4.4.2. Determination of Hydroxyl Radical Scavenging Capacity

Referring to the method of Xia et al. [53], 8.8 mmol/L H_2_O_2_, 0.5 mL 9 mmol/L Fe^2+^, and 0.5 mL 9 mmol/L salicylic acid-ethanol solutions were added to 100 μL of different concentrations of MLPH. Distilled water was used as the blank Group *A*_0_. The absorbance value *A*_1_ at each concentration was measured at 510 nm, and the hydroxyl radical scavenging activity equation was as follows.

(3) $$\mathrm{Hydroxyl}\;\mathrm{radical}\;\mathrm{scavenging}\;\mathrm{activity}\;(\%)=\frac{A_{0}-A_{1}}{A_{0}}\times 100,$$

where *A*_0_ is the light absorption value of the sample group, and *A*_1_ is the light absorption value of the blank group.

#### 4.4.3. Superoxide Anion Radical Scavenging Capacity

Referring to the method of Miao et al. [54], 2.25 mL of Tris-HCl buffer (0.1 mol/L) with pH 8.2 was added to l mL of distilled water and 1 mL of various concentrations of sample solution, and then 0.25 mL of 10 mmol/L o-triphenol was added. After mixing well, the reaction was heated in a water bath at 25 °C, the reaction was aborted by adding 20 μL of HCl at the 3rd min, and the absorbance value was measured at 325 nm. The clearances were calculated as follows.

(4) $$\mathrm{Superoxide}\;\mathrm{anion}\;\mathrm{radical}\;\mathrm{scavenging}\;\mathrm{activity}\;(\%)=\frac{A_{0}-(A_{1}-A_{2})}{A_{0}}\times 100,$$

where *A*_0_ is the absorbance value of the blank sample with distilled water instead of the sample solution; *A*_1_ is the absorbance value of the sample solution; and *A*_2_ is the absorbance value of the solution without catechol.

#### 4.4.4. ABTS Radical Scavenging Capacity

The ABTS radical scavenging activity was referred to the method of Yazdi et al. [55] and Hu et al. [56] with appropriate modifications. First, 2.45 mmol/L potassium persulfate was mixed in a 1:1 ratio with 7 mmol/L ABTS mother liquor, and the reaction was carried out for 12–16 h. Next, the samples were diluted with 5 mM pH 7.4 PBS to achieve an absorbance value of 0.7~0.8 at 734 nm, and finally, the samples were mixed 1:1 with the diluted ABTS free radical working solution, and the reaction was carried out for 10 min at room temperature while protected from light. The calculation formula is as follows.

(5) $$\mathrm{ABTS}\;\mathrm{radical}\;\mathrm{scavenging}\;\mathrm{activity}\;(\%)=\frac{A_{0}-A_{1}}{A_{0}}\times 100,$$

where *A*_0_ is the absorbance value of the blank group, and *A*_1_ is the absorbance value of the sample group.

### 4.5. Detection of α-Glucosidase Inhibitory Activity of MOLP

#### 4.5.1. Determination of the Reaction Time of α-Glucosidase with the Substrate PNPG

Three parallels were made for each time group according to the method studied by Shen [57]. Firstly, 40 μL of 0.01 mol/L PBS buffer solution (without 0.2% BSA) and 20 μL of 0.3 U/mL α-glucosidase solution were added to a 96-well plate and incubated for 5 min in an incubator at 37 °C. Then, 20 μL of 2.5 mmol/L PNPG solution was added and placed in the incubator for 20, 25, 30, 35, and 40 min, respectively. Eighty μL of 0.2 mol/L Na_2_CO_3_ solution was added to terminate the reaction. The final absorbance at 405 nm was measured and plotted, and the most appropriate reaction time was selected.

#### 4.5.2. α-Glucosidase Activity Assay

The sample group, background group, negative group, and blank group were set according to the method of Yang et al. [33], with a slight change. Sample solutions were prepared at concentrations of 0.1, 0.2, 0.4, 0.8, 1.6, and 3.2 mg/mL. For the sample group, 20 μL of 0.01 mol/L PBS buffer solution (without 0.2% BSA), 20 μL of different concentrations of sample solution, and 20 μL of α-glucosidase were added; for the background group, 40 μL of 0.01 mol/L PBS buffer solution (without 0.2% BSA) and 20 μL of different sample solutions were added; for the negative group, 40 μL of 0.01 mol/L PBS buffer solution (without 0.2% BSA) and 20 μL of α-glucosidase solution to the negative group; 80 μL of 0.01 mol/L PBS buffer solution (without 0.2% BSA) to the blank group. The reaction was incubated in an incubator at 37 °C for 10 min and then taken out. All except for the blank group were added 20 μL of PNPG solution and continued to incubate for 35 min, removed, and terminated by adding 80 μL of 0.2 mol/L Na_2_CO_3_ solution. Three parallel experiments were done for each group and the absorbance was measured at 405 nm using an enzyme marker. The calculation formula is as follows.

A-glucosidase inhibition (%) = 1 − (OD sample − OD background)/(OD negative − OD blank) × 100%.(6)

#### 4.5.3. Kinetic Analysis of the Inhibitory Effect of MOLP on α-Glucosidase Activity

PNPG solutions were configured at concentrations of 0.2, 0.5, 2, 5, and 10 mmol/mL and fixed alpha-glucose concentrations of 0.3 U/mL. The sample solution concentrations as well as the assay were consistent with Section 4.5.2. The curves were plotted in horizontal (1/PNPG) and vertical (1/V) coordinates according to the Lineweaver–Burk equation and the type of inhibition of the reaction was determined.

#### 4.5.4. Effect of MOLP on Insulin-Resistant HepG2 Cells

##### MTT Cell Viability Assay

HepG2 cells were cultured at 37 °C in a humidified environment with 5% CO_2_. All media were supplemented with 10% foetal bovine serum and 1% penicillin-streptomycin mixture. HepG2 cells in logarithmic growth phase were inoculated in 96-well plates and adjusted to a concentration of 1 × 10^4^ cells per well. Experimental group cells were treated with different concentrations of MOLP (100, 200, 300, 400, 500, 600, 700, 800, and 900 μg/mL). MOLP was added to the blank group without the addition of HepG2 cells. After 24 h of incubation, 5 μg/mL MTT was added to each well for 4 h. The medium was replaced with DMSO, and the OD was measured at a wavelength of 490 nm by a microplate reader. The cell survival rate was calculated by this formula:

Survival rate of HepG2 cells (%) = (OD experimental group − OD blank group)/(OD control group − OD blank group) × 100%

##### Effect of MOLP on Glucose Consumption and Glycogen Synthesis in IR-HepG2

After 24 h of HepG2 cell culture, 200 μL of suspension was adsorbed and inoculated into 96-well plates at a density of 1 × 10^4^/well, grouped into control, protection, and insulin resistance groups. The insulin-resistant group was prepared according to the method of Song et al. [58] as follows: 30 mM glucose was added and applied to HepG2 cells for 24 h, followed by incubation with 100 nM insulin for 10 min. For the protection group, the following steps were used on top of the insulin resistant group: MOLP (100, 200, and 300 μg/mL) DMEM solution and metformin (MC, 100 μg/mL) DMEM solution were added and incubated for 24 h. The control group was incubated for 48 h in 200 μL of medium; The blank group was incubated for 48 h with MOLP added without the addition of HepG2 cells. Glucose consumption and glycogen synthesis was measured using glucose kits and glycogen reagents.

##### Effect of MOLP on CAT, SOD, GSH-Px, MDA and SOD in IR-HepG2 Cells

HepG2 cells (3 × 10^5^) were pipetted separately in 6 (60 mm) cell culture dishes and were cultured for 24 h. After performing different treatments (the experimental treatment is the same as in Section 4.5.4), total superoxide dismutase (SOD) and catalase (CAT) and glutathione peroxidase (GSH-Px) activities and malondialdehyde (MDA) contents in cell lysis products were determined according to the method in the SOD, CAT, GSH-Px, and MDA kit instructions.

The Reactive Oxygen Assay Kit is a kit used for the detection of reactive oxygen species using the fluorescent probe H2DCFDA. H2DCFDA itself is non-fluorescent and can freely pass through the cell membrane. Once inside the cell, it can be hydrolysed by intracellular esterases to generate DCFH, which is not permeable to the cell membrane, thus allowing the probe to be easily loaded into the cell. The level of intracellular reactive oxygen species can be determined by measuring the fluorescence of DCFH, which oxidises non-fluorescent DCFH to produce fluorescent DCF. Based on the production of fluorescence in living cells, the level and variation of cellular reactive oxygen species can be determined. Therefore, ROS were measured using the Reactive Oxygen Species Assay Kit, HepG2 cells were inoculated with 2 × 10^5^ cells/well in laser confocal dishes in combination with the method of Ladda et al. [59], and the cells were treated according to the experimental grouping method in Section 4.5.4. Pictures were taken with a laser confocal microscope, and fluorescence intensity analysis was performed by ImageJ.

### 4.6. Statistical Analysis

The data obtained in this study were statistically analysed using ANOVA by GraphPad Prism 8 (GraphPad Software, Inc., San Diego, CA, USA). All experimental results are expressed as mean ± standard deviation (SD), with significant differences compared to the insulin-resistant group (* *p* < 0.05, ** *p* < 0.01, *** *p* < 0.001).

## 5. Conclusions

In summary, this study achieved (17.03 ± 1.03)% extraction of *Moringa oleifera* leaves polysaccharides by ultrasonic assisted cellulase with response surface optimisation of the extraction process. The extracted MOLP showed some inhibitory effect on α-glucosidase, significantly alleviated insulin resistance, and reduced oxidative stress in IR-HepG2 cells. This work provides a theoretical basis for the optimization of the extraction process of *Moringa oleifera* polysaccharide and a reference for further exploration of the application of *Moringa oleifera* polysaccharide in antioxidant and glucose regulation.

## Figures and Tables

**Table 1:** Results of the Plackett–Burman test.

Std	Run	*A* The Additive Quantity of Cellulase (%)	*B* Hydrolysis Temperature (°C)	*C* Hydrolysis Time (°C)	*D* pH	*E* Ultrasonic Power (W)	*F* Ultrasonic Temperature (°C)	*G* Ultrasound Time (min)	*H* Ratio of Material to Water (g/mL)	Extraction Yield (%)
11	1	0.9	55	150	7	540	55	20	20	16.39 ± 0.34
4	2	0.7	65	90	7	540	55	40	40	17.25 ± 0.53
5	3	0.7	55	150	6	540	65	20	40	15.05 ± 0.23
3	4	0.9	55	150	7	420	65	40	40	15.46 ± 0.54
8	5	0.9	65	90	6	420	65	20	40	16.71 ± 0.46
1	6	0.9	65	90	7	540	65	20	20	16.98 ± 0.68
9	7	0.9	65	150	6	420	55	40	20	16.91 ± 0.79
2	8	0.7	65	150	6	540	65	40	20	17.81 ± 0.65
12	9	0.7	55	90	6	420	55	20	20	14.21 ± 0.84
6	10	0.7	55	90	7	420	65	40	20	14.21 ± 0.32
7	11	0.9	55	90	6	540	55	40	40	17.81 ± 0.42
10	12	0.7	65	150	7	420	55	20	40	14.29 ± 0.36

**Table 2:** Plackett–Burman test ANOVA.

Source	Sum of Square	df	Mean Square	*F* Value	*p* Value	
Model	20.28	8	2.54	10.75	0.0382	*
*A* The additive quantity of cellulase (%)	4.61	1	4.61	19.53	0.0215	*
*B* Hydrolysis temperature (°C)	3.88	1	3.88	16.47	0.027	*
*C* Hydrolysis time (°C)	0.13	1	0.13	0.54	0.5155	
*D* pH	1.28	1	1.28	5.41	0.1024	
*E* Ultrasonic power (W)	7.53	1	7.53	31.92	0.011	*
*F* Ultrasonic temperature (°C)	0.036	1	0.036	0.15	0.7233	
*G* Ultrasonic time (min)	2.83	1	2.83	11.98	0.0406	*
*H* Ratio of material to water (g/mL)	4.38 × 10^−4^	1	4.38 × 10^−4^	1.86 × 10^−3^	0.9683	
Residual	0.71	3	0.24			
Cor Total	20.99	11				

Note: *p* < 0.05 *.

**Table 3:** Box–Behnken test results.

Std	Run	*A* The Additive Quantity of Cellulase (%)	*B* Hydrolysis Temperature (°C)	*C* Ultrasonic Power (W)	*D* Ultrasonic Time (min)	Extraction Yield (%)
19	1	0.7	60	540	30	15.47 ± 0.32
18	2	0.9	60	420	30	15.50 ± 0.65
25	3	0.8	60	480	30	17.03 ± 0.75
28	4	0.8	60	480	30	17.71 ± 0.32
26	5	0.8	60	480	30	17.67 ± 0.65
6	6	0.8	60	540	20	15.11 ± 0.12
20	7	0.9	60	540	30	15.45 ± 0.35
17	8	0.7	60	420	30	15.41 ± 1.12
7	9	0.8	60	420	40	15.62 ± 0.23
22	10	0.8	65	480	20	14.71 ± 0.56
27	11	0.8	60	480	30	17.54 ± 0.31
3	12	0.7	65	480	30	15.17 ± 0.43
15	13	0.8	55	540	30	16.02 ± 0.32
29	14	0.8	60	480	30	17.90 ± 0.68
4	15	0.9	65	480	30	15.32 ± 0.78
10	16	0.9	60	480	20	15.41 ± 0.36
2	17	0.9	55	480	30	15.90 ± 1.2
9	18	0.7	60	480	20	13.77 ± 0.86
23	19	0.8	55	480	40	14.40 ± 0.35
11	20	0.7	60	480	40	14.67 ± 0.65
21	21	0.8	55	480	20	15.55 ± 0.67
1	22	0.7	55	480	30	15.38 ± 0.75
13	23	0.8	55	420	30	14.16 ± 0.36
12	24	0.9	60	480	40	16.09 ± 0.53
8	25	0.8	60	540	40	15.89 ± 0.63
14	26	0.8	65	420	30	15.33 ± 0.54
16	27	0.8	65	540	30	15.18 ± 0.63
5	28	0.8	60	420	20	14.17 ± 0.35
24	29	0.8	65	480	40	15.80 ± 0.45

**Table 4:** Analysis of variance for the Box–Behnken regression model.

Source	Sum of Square	df	Mean Square	*F* Value	*p* Value	
Model	28.7	14	2.05	8.71	0.0001	***
*A* The additive quantity of cellulase	1.21	1	1.21	5.15	0.0396	*
*B* Hydrolysis temperature	1.15 × 10^−3^	1	1.15 × 10^−3^	4.88 × 10^−3^	0.9453	
*C* Ultrasonic power	0.72	1	0.72	3.04	0.1032	
*D* Ultrasonic time	1.18	1	1.18	5.01	0.0419	*
*AB*	0.034	1	0.034	0.14	0.7117	
*AC*	3.11 × 10^−3^	1	3.11 × 10^−3^	0.013	0.9102	
*AD*	0.013	1	0.013	0.054	0.8196	
*BC*	1.01	1	1.01	4.28	0.0577	
*BD*	1.27	1	1.27	5.38	0.036	*
*CD*	0.11	1	0.11	0.48	0.5003	
*A* ^2^	7.44	1	7.44	31.59	<0.0001	
*B* ^2^	8.58	1	8.58	36.43	<0.0001	
*C* ^2^	7.88	1	7.88	33.48	<0.0001	
*D* ^2^	12.09	1	12.09	51.33	<0.0001	
Residual	3.3	14	0.24			
Lack of Fit	2.86	10	0.29	2.66	0.1796	
Pure Error	0.43	4	0.11			
Cor Total	32	28				

Note: R^2^ = 0.897, *p* < 0.05 *; *p*< 0.001 ***.

**Table 5:** Ultrasound-assisted cellulase extraction of *Moringa oleifera* polysaccharides one-way experimental design.

Independent Variables	Levels				
The additive quantity of cellulase (%)	0.6	0.7	0.8	0.9	1.0
Hydrolysis temperature (°C)	50	55	60	65	70
Hydrolysis time (°C)	30	60	90	120	150
pH	5	5.5	6	6.5	7
Ultrasonic power (W)	300	360	420	480	540
Ultrasonic temperature (°C)	55	60	65	70	75
Ultrasound time (min)	10	20	30	40	50
Ratio of material to water (g/mL)	1:20	1:30	1:40	1:50	1:60

## Supplementary Materials

The supporting information can be downloaded at: https://www.mdpi.com/article/10.3390/ijms232012405/s1.
