# Supplementary material for: Ultrasonic-Cellulase Synergistic Extraction of Crude Polysaccharides from Moringa oleifera Leaves and Alleviation of Insulin Resistance in HepG2 Cells
Source: Int J Mol Sci. 2022 Oct 17;23(20):12405. doi: 10.3390/ijms232012405 (PMC9604441; doi:10.3390/ijms232012405)
Supplement: Supplementary file 1 [file ijms-23-12405-s001.zip › ijms-1915835-supplementary.pdf]

Ultrasonic-cellulase synergistic extraction of crude polysaccharides from *Moringa oleifera* leaves and alleviation of insulin resistance in HepG2 cells

Fan Gu [1,2], Liang Tao [1,2,3,*], Runling Chen [1], Jiao Zhang [1], Xingzhong Wu [1], Min Yang [1,2], Jun Sheng [4] and Yang Tian [1,2,3,*]

[1] College of Food Science and Technology, Yunnan Agricultural University, Kunming 650201, China
[2] National Research and Development Professional Center for Moringa Processing Technology, Yunnan Agricultural University, Kunming 650201, China
[3] Engineering Research Center of Development and Utilization of Food and Drug Homologous Resources, Ministry of Education, Yunnan Agricultural University, Kunming 650201, China
[4] Yunnan Provincial Engineering Research Center for Edible and Medicinal Homologous Functional Food, Yunnan Agricultural University, Kunming 650201, China
* Correspondence: 2017043@ynau.edu.cn (L.T.); tianyang@ynau.edu.cn (Y.T.)

Supplementary Data

S1 Glucose standard curve

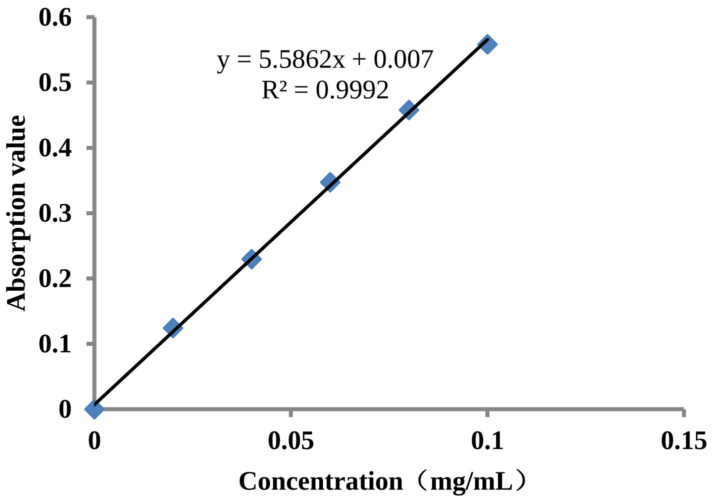

Figure S1 Glucose standard curve

S2. Molecular weight analysis

(1) Method

The homogeneity and molecular weight of the various fractions were measured using SEC-MALLS-RI. The weight Mw of various fractions in 0.1 M NaNO3 aqueous solution containing 0.02% NaN3 (or DMSO solution containing 0.5% LiBr) was measured on a DAWN HELEOS-II laser photometer (Wyatt Technology Co, three tandem columns (300 × 8 mm, Shodex OH-pak SB-805, 804 and 803; Showa Denko K.K., Tokyo, Japan) using Sanshu Biotech. Ltd. (Shanghai, China). The flow rate was 0.4 mL/min (or 0.3 mL/min). A

differential refractive index detector (Optilab T-rEX, Wyatt Technology Co., USA) was also connected to give the concentration and dn/dc values of the fractions. The dn/dc value of the fraction was determined to be 0.141 mL/g in 0.1 M NaNO3 aqueous solution containing 0.02% NaN3 and 0.07 mL/g in DMSO solution. data were acquired and processed using ASTRA 6.1 (Wyatt Technology).

(2) Results

Mn (kDa)	Uncertainty	Mp (kDa)	Uncertainty	Mw (kDa)	Uncertainty	Mz (kDa)	Uncertainty
12.244	0.01127	4.481	0.0115	279.448	0.00828	1865.07	0.02188

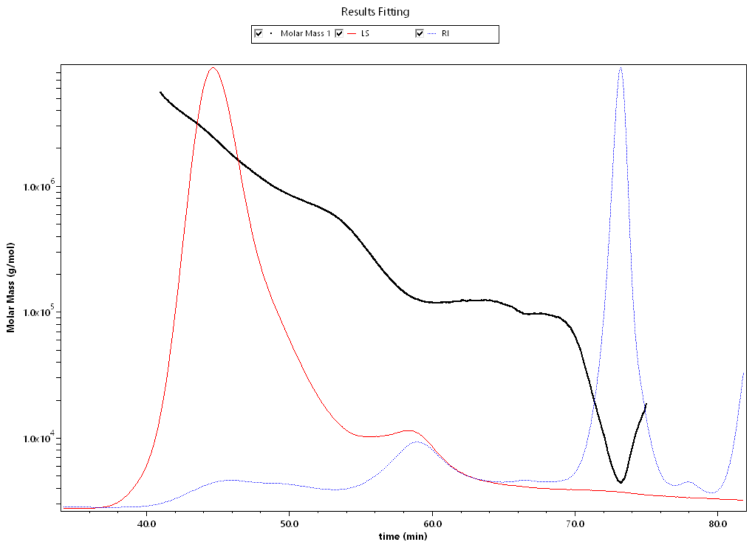

Figure S2 Molecular weight diagram of crude polysaccharides from *Moringa oleifera* leaves

S3 Process optimisation

Table S1 Table of Factor Levels for PB Experimental Design

Factors	Level		
	-1	0	1
A The additive quantity of cellulase（%）	0.7	0.8	0.9
B Hydrolysis temperature（℃）	55	60	65
C Hydrolysis time（℃）	90	120	150
D pH	6	6.5	7
E Ultrasonic power (W)	420	480	540
F Ultrasonic temperature（℃）	55	60	65
G Ultrasound time （min）	20	30	40
H Ratio of material to water (g/mL)	1:20	1:30	1:40

Table S2 Level and code of independent variables used for response surface analysis

Factors	Level		
	-1	0	1
A The additive quantity of cellulase	0.7	0.8	0.9
B Hydrolysis temperature	55	60	65
C Ultrasonic power	420	480	540
D Ultrasound time	20	30	40

S4 Crude polysaccharide monosaccharide composition of *Moringa oleifera* leaves

Table S3 The monosaccharide composition of MOPL

monosaccharide	Fuc	Rha	Ara	Gal	Glc	Xyl	Man	Gal-UA	Glc-UA
%	0.52%	10.52%	24.86%	49.88%	5.67%	2.05%	0.92%	4.35%	1.24%

(1) Method

The monosaccharide composition was analysed by the PMP-HPLC method. Briefly, the sample (1-2 mg) was completely hydrolysed with 2 M trifluoroacetic acid (TFA) at 110 °C for 4 h. The hydrolysis product was dried under vacuum and then derivatised with 50 μL of 1-phenyl-3-methyl-5-pyrazolone (PMP) solution (0.5 M methanol solution) and 50 μL of 0.6 M NaOH at 70 °C for 100 min. The reaction was terminated by neutralization with 100 μL of 0.3 M HCl and 900 μL of distilled water, followed by extraction with chloroform (1 mL, 3 times). The extracts were analysed by HPLC on a Fortis C18 column (5 μm, 4.6 × 250 mm) on an Agilent 1200 instrument equipped with a G1362A Binpump and a G1314F UV detector.

(2) Results

The monosaccharide composition of MOLP is shown in Table S2. galactose (49.88%), arabinose (24.86%) and rhamnose (10.52%) were the major components, while rock sugar (0.52%), glucose (5.67%), xylose (2.05%), mannose (0.92%), galacturonic acid (4.35%) and glucuronic acid (1.24) were the with small amounts of residues.
